# OH End-Capped Silicone as an Effective Nucleating Agent for Polylactide—A Robotizing Method for Evaluating the Mechanical Characteristics of PLA/Silicone Blends

**DOI:** 10.3390/polym16081142

**Published:** 2024-04-18

**Authors:** Robert E. Przekop, Bogna Sztorch, Julia Głowacka, Agnieszka Martyła, Eliza Romańczuk-Ruszuk, Marek Jałbrzykowski, Łukasz Derpeński

**Affiliations:** 1 Centre for Advanced Technologies, Adam Mickiewicz University in Poznań, 10 Uniwersytetu Poznańskiego, 61-614 Poznań, Poland; rprzekop@amu.edu.pl (R.E.P.); julia.glowacka@amu.edu.pl (J.G.); agnieszka.martyla@amu.edu.pl (A.M.); 2 Faculty of Chemistry, Adam Mickiewicz University in Poznań, 8 Uniwersytetu Poznańskiego, 61-614 Poznań, Poland; 3 Institute of Biomedical Engineering, Faculty of Mechanical Engineering, Bialystok University of Technology, Wiejska 45C Street, 15-351 Bialystok, Poland; e.romanczuk@pb.edu.pl; 4 Institute of Mechanical Engineering, Faculty of Mechanical Engineering, Bialystok University of Technology, Wiejska 45C Street, 15-351 Bialystok, Poland; m.jalbrzykowski@pb.edu.pl

**Keywords:** polylactide (PLA), polysiloxane, nucleating agent, crystallization, automation, mechanical properties, AI feeding data, high-throughput methods

## Abstract

Current research on materials engineering focuses mainly on bio-based materials. One of the most frequently studied materials in this group is polylactide (PLA), which is a polymer derived from starch. PLA does not have a negative impact on the natural environment and additionally, it possesses properties comparable to those of industrial polymers. The aim of the work was to investigate the potential of organosilicon compounds as modifiers of the mechanical and rheological properties of PLA, as well as to develop a new method for conducting mechanical property tests through innovative high-throughput technologies. Precise dosing methods were utilized to create PLA/silicone polymer blends with varying mass contents, allowing for continuous characterization of the produced blends. To automate bending tests and achieve comprehensive characterization of the blends, a self-created workstation setup has been used. The tensile properties of selected blend compositions were tested, and their ability to withstand dynamic loads was studied. The blends were characterized through various methods, including rheological (MFI), X-ray (XRD), spectroscopic (FTIR), and thermal properties analysis (TG, DSC, HDT), and they were evaluated using microscopic methods (MO, SEM) to examine their structures.

## 1. Introduction

Research into bio-based materials is one of the main streams currently being developed in materials engineering. Among the most promising starch-derived polymers is polylactide (PLA), whose natural properties do not differ significantly from those of polymers such as acrylonitrile butadiene styrene (ABS), polyamide (PA), or polycarbonate (PC) used in industrial applications. It has a tensile strength of 30–70 MPa, depending on the PLA grade and forming technology used, which is similar to that of technical ABS (45–70 MPa), PC (30–70 MPa), and PA6 (50–80 MPa). Additionally, it has a plasticization temperature of approximately 165 °C, making it suitable for injection molding, extrusion, and additive manufacturing processing [1,2,3].

This semicrystalline polyester material offers promising feature set properties and could be considered as an alternative to petrochemical polymers, derived from depleting fossil resources. Polylactide is a fully compostable and biodegradable type of plastic. When it decomposes, it turns into carbon dioxide and water, without leaving any harmful residue in the environment [4]. Other examples of biodegradable polymers similar to PLA include TPS (thermoplastic starch) [5] and PCL (polycaprolactone) [6]. However, thermoplastic starch is typically used as an additive due to its low mechanical properties in its pure form. On the other hand, PCL has a low plasticization temperature (around 60 °C), which limits its applications. In comparison, polylactide is a more favorable material. Due to its sensitivity to external factors, such as mechanical stress (especially low-impact durability), PLA is not suitable for long-term applications or for producing critical device components. Various additives are used to modify its properties and create PLA-based compounds. However, adding these additives can negatively affect other performance characteristics, so finding the best PLA blend configurations and compatible additives is important. To fully explore its potential, additional research is necessary to better understand its characteristics and possible applications [7].

There are various methods of modifying polymers such as PLA to enhance their properties. These methods include plasticization, to make it more flexible, pliable, and easier to work with through the use of different types of ester compounds, e.g., various citrate esters [8,9], bis(2-ethyl hexyl) adipate (DOA) [10], limonene derivatives [11], polyethylene glycol (PEG) [12], and oligomeric lactic acid (OLA) [13]; the use of nucleating agents to promote the formation of crystalline structures and improve dimensional stability, and its barrier and mechanical properties [14,15,16]; adding mineral fillers, e.g., silica [17], lake sediments [18], clays [19], titanium dioxide [20], and diatomaceous earth [21], to enhance mechanical performance and lower the composite’s total cost; and blending with other polymers, e.g., thermoplastic starch (TPS) [22,23], thermoplastic polyurethanes (TPU) [24,25], poly(butylene succinate-co-adipate) (PBSA) [26,27], different varieties of polyamides (PA) [28,29], and natural rubbers [30,31] to make it more flexible. Unfortunately, the improvement of one parameter often harms other PLA properties, so further research into improving the performance properties of this environmentally friendly polymer is needed. Optimizing the performance characteristics of such materials is a complex undertaking that requires a thorough understanding of the interplay between their various properties.

Organosilicone polymers (silicones, polysiloxanes) are macromolecular compounds that, due to their unique inorganic-organic structure, exhibit several different properties compared to polymers with a carbon main chain. They have better thermal stability over a wide range of temperatures due to the partially ionic nature of the Si–O–Si bond between the main chain elements, are highly flexible, show greater resistance to environmental factors (UV, moisture, gamma radiation), and can therefore be an interesting additive to polylactide [32]. They are environmentally safe, and are mainly used in biomedical engineering [33] but also as additives in cosmetics [34] and lubricants [35]. Their use to modify polylactide will not carry negative environmental impacts, which fits in with the requirements of the circular economy by minimizing the negative effects resulting from the decomposition of solid polymer waste in the environment and the use of green components.

The literature offers several research perspectives regarding the use of polysiloxanes in PLA blends. Researchers explore different crosslinking techniques to enhance the compatibility between PLA and silicone polymers. Meekum Utai and Apichart Khiansanoi have conducted extensive research on the development of crosslinking multicomponent systems incorporating polylactide, silicone, polyols, and silanes in the presence of curing agents such as TETA (triethylenetetramine). Their findings demonstrate how the composition of different components affects mechanical, rheological, morphological, and heat distortion temperatures (HDT) [36]. In a similar study, Utai and Khiansanoi discussed PLA and single-component silicone rubber blends for sub-zero temperature applications. They proposed PLA/RTV silicone single-component polymer systems, with the addition of silane, polyol, and talc coagents crosslinked by sauna curing. The resulting macro chain crosslinks via the silane/moisture condensation mechanism led to increased viscous behavior, trimmed by the creation of intermolecular chain architecture. This, in turn, enhanced impact toughness at sub-zero temperatures [37]. Yıldız Sibel, Bağdagül Karaağaç, and Guralp Ozkoc focused their efforts on PLA/vinyl terminated silicone rubber blends prepared via dynamic crosslinking in the presence of DCP (dicumyl peroxide) and the coagent TAC (triallyl cyanurate). Their study demonstrated the effectiveness of silicone rubber in toughening PLA, resulting in a significant improvement in impact strength. However, the incorporation of silicone rubber led to a deterioration in the yield strength and stiffness of the blends. The study also highlighted the positive impact of decreasing the cold crystallization temperature of PLA, and included a morphology study [38]. Khuenkeao Thidarat, Nawadon Petchwattana, and Sirijutaratana Covavisaruch described the production of PLA films with improved ductility and elastic properties, incorporating ultrafine vulcanized silicone rubber and talc. Their findings show how these components influenced PLA films’ thermal and tensile properties [39].

High-throughput methods (HTM) are powerful tools that can significantly improve technical processes and greatly facilitate the day-to-day work of research laboratories. However, despite their potential benefits, this approach to experimentation and research planning is still met with resistance from the academic community and small R&D companies with limited capital. This resistance is due to the prevailing perception that such methods are inadequate for fundamental research, involve significant financial expenditure, and are not suitable for developing materials with complex physicochemical properties, given the complexity of the design of experiments (DoE) in the context of materials engineering [40]. With the increasing automation and robotization of processes and the development of new user-friendly software tools, researchers are now showing a greater interest in high-throughput methods of materials testing and development. These methods are valued for their versatility and their ability to rapidly deliver the large amounts of data they can provide, which is crucial considering today’s urgent need for better materials with advanced and specified properties [41]. High-throughput experimental methods (HTE) and high-throughput tools (HTT) are of particular interest in fields such as drug design [42], research into new biomaterials [43], biology [44], and biotechnology [45], and they were also used in the design of metallic superalloys [46].

The objective of our investigation is to assess the potential of utilizing organosilicon polymers as effective modifiers to enhance the mechanical and rheological traits of polylactide. Furthermore, we introduce a new method for performing mechanical property examinations using advanced high-throughput technologies. In this work, simple test equipment, through modification of traditional research equipment and robotic tools, was developed. It requires a minimal investment, but it significantly increases the effectiveness of the three-point bending testing process. Additionally, it allows for a thorough, continuous analysis of the properties of the created polymeric materials. Our previous research has indicated the advantageous impacts of organosilicon compounds on the physicochemical and mechanical characteristics of not only polylactide, but also other types of polymers [47,48,49]. In this study, blends of PLA (polylactic acid) and silicone polymer, with varying mass content of each component, were produced using precise dosing methods. The blends were continuously characterized, and selected samples were evaluated for their tensile properties and resistance to dynamic loading. The blends were also subjected to various tests such as rheological (MFI), X-ray (XRD), and spectroscopic (FTIR) evaluations, as well as thermal (TG, DSC, HDT) analyses and microscopic assessment using MO and SEM methods, to determine their structure and properties.

## 2. Materials and Methods

### 2.1. Materials

Polylactide (PLA) Ingeo 2003D type was purchased from NatureWorks (Minnetonka, Minneapolis, MN, USA). OH end-capped silicone fluid, type BRB Flexosil OH Fluid 20,000 (viscosity = 20,000 cSt), was purchased from BRB International B.V. (Ittervoort, The Netherlands).

### 2.2. Preparation of PLA/Silicone Blends

PLA/silicone blends were prepared using a ZAMAK MERCATOR WG 150/280 (Zamak Mercator, Skawina, Poland) laboratory two-roll mill. For the preparation of the 15% masterbatch, 1700 g of PLA Ingeo™ 2003 D and 300 g of silicone OH Fluid 20,000 were employed. The blending components were mixed for 15 min at 215 °C, and the speed of the rollers was 20 rpm. The process was carried out until full homogenization of the components was achieved. The resulting polymer system was then granulated using a SHINI SG-1417-CE grinding mill and dried at 55 °C/24 h.

### 2.3. Preparation of Final Samples

The finished 15% PLA/silicone masterbatch was used to obtain shapes with a lower concentration of silicone in PLA on an Engel E-victory170/80 injection molding machine. Table S1 shows the injection molding parameters. The mold temperature was maintained at room temperature. A holding pressure with a linear increment over time was applied. Standardized specimens for mechanical testing, in accordance with PN-EN ISO 20753:2019-01 [50], were fabricated. Beams for automated robotic flexural tests were injected in a continuous feed system with a dosage accuracy of 0.5% in order to obtain a comprehensive characterization of the material over a range of silicone concentrations from 0 to 15%. Standard 1A strength specimens were injected for selected concentrations of 0.5%, 1.0%, 2.5%, 5.0%, 10%, and 15% (Table 1).

### 2.4. Experimental Workstation Setup Design for Automated Robotic Flexural Tests

A special test ring was set up to automate the flexural tests (Figure S1). The operation of the workstation and its components was presented in more detail in our previously published work [51]. In the presented bench solution, a modification was made to the two-part specimen holder by increasing the height of the front face of its lower grip section. This improvement created a larger surface area for the specimen bulldozer, preventing the specimen from overlapping or mis-positioning during testing. Rubber inserts were also added to the gripping surfaces for better specimen stability when using the robotic arm.

### 2.5. Characterization Methods

For automatized robotic flexural tests, standardized type B specimens were used. The tests were performed using an MTS Insight testing machine (MTS Systems Corporation, EdenPrairie, MN, USA). The servo-mechanical testing machine allows for experimental testing under axial loads of up to 1 kN and an elongation/flexural range of up to 750 mm. The traverse speed for the measurements was set at 2 mm/min. The measurement was carried out until a deflection arrow of 15 mm was achieved or the specimen broke. The automatic test was conducted continuously over a concentration range of 0 to 15% silicone content in the polymer blends. A total of 203 samples were fluently tested.

Flexural tests in manual mode were performed on an INSTRON 5969 universal testing machine with a maximum load force of 50 kN (Instron, Norwood, MA, USA). The specimens were prepared according to the requirements of PN-EN ISO 178 [52]. The traverse speed for measurement was set at 2 mm/min.

For tensile strength tests, standard 1A specimens were used, following the requirements of PN-EN ISO 527 [53]. Tests of the obtained specimens were performed on an INSTRON 5969 universal testing machine with a maximum load force of 50 kN (Instron, Norwood, MA, USA). The traverse speed for measurement was set at 5 mm/min. For all the series, seven measurements were performed. The average and standard deviation were determined for each measurement series.

A Charpy impact test (with no notch) was performed on an Instron Ceast 9050 impact machine (Instron, Norwood, MA, USA), according to PN-EN ISO 179 [54]. For all the series, seven measurements were performed. The average and standard deviation were determined for each measurement series.

The effect of the modifier addition on the mass flow rate (MFR) was also determined. The measurements were performed using an Instron plastometer (Norwood, MA, USA), model Ceast MF20 (Instron, Norwood, MA, USA), according to the applicable standard PN-EN ISO 1133 [55]. The measurement temperature was 210 ± 0.5 °C, while the piston loading was 2.16 kg.

Thermogravimetry (TG) was performed using a NETZSCH 209 F1 Libra gravimetric analyzer (Selb, Germany). Samples of 5 ± 0.2 mg were cut from each granulate and placed in Al_2_O_3_ crucibles. Measurements were conducted under nitrogen (flow of 20 mL/min) in the range of 30–950 °C at a 10 °C/min heating rate. Differential scanning calorimetry (DSC) was performed using a NETZSCH 204 F1 Phoenix calorimeter (Selb, Germany). Samples of 5 ± 0.2 mg were cut from each granulate and placed in an aluminum crucible with a punctured lid. The measurements were performed under nitrogen in the temperature range of 20–200 °C and at a 10 °C/min heating rate. The crystallinity level (*Xc*) was calculated using the following Formula (1), according to the method included in the literature [56]:

(1) $$X_{c}=\frac{∆H_{M}}{\left(1-\varphi \right)\cdot ∆H_{MPLA}}\cdot 100\%$$

where:

∆ *H_M_*—melt enthalpy;
∆ *H*cc—cold crystallization enthalpy;
∆ *H_MPLA_*—melting enthalpy corresponding to crystalline PLA 93 J/g [57];
*φ*—silicone amount in the blend material.

Heat distortion temperature (HDT) tests were carried out on specimens with dimensions corresponding to the flexural beam (4 × 10 × 80 mm), and the test was carried out in accordance with PN-EN ISO 75 [58], HDT-A (1.8 MPa). For all measurement series, three measurements each were obtained, and the result was averaged.

X-ray diffraction (XRD) was performed using a powder diffractometer (SmartLab Rigaku, Japan) with a CuK alpha lamp, in the range of 3–100 (2 thetas), with a scan step of 0.01 and a scan speed 4°/min. Every 20th sample after the flexural test was investigated using XRD, which corresponds to a 2% change in the concentration of silicone in the blend in an individual sample. The crystallinity level (*X_c_*) was calculated using the following Formula (2):

(2) $$X_{c}=\frac{A_{C}}{\left(A_{C}+A_{a}\right)}$$

where:

*X_c_* [%]—degree of crystallinity;
*A_c_*—crystallized area on the diffractogram;
*A_a_*—amorphous area on the diffractogram.

The Fourier-transform infrared (FTIR) spectra were recorded on a Nicolet iS 50 Fourier transform spectrophotometer (Thermo Fisher Scientific, Waltham, MA, USA) equipped with a diamond ATR (attenuated total reflection) unit with a resolution of 0.09 cm^−1^.

Scanning electron microscopy (SEM) microphotographs were captured using a Quanta FEG 250 (FEI) high-resolution scanning electron microscope (Thermo Fisher Scientific, Waltham, MA, USA) to analyze the microstructure and quality of the produced samples. Scanning electron microscopy with energy dispersive spectroscopy (SEM/EDS) analyses were also recorded on a Quanta FEG 250 (FEI) instrument (Thermo Fisher Scientific, Waltham, MA, USA), with SEM at 5 kV and EDS at 30 kV.

The surface topography was analyzed using a Digital Light Microscope Keyence VHX 7000 with 100× to 1000× VH-Z100T lens (Osaka, Japan). All of the images were recorded with a VHX 7020 camera.

## 3. Results

### 3.1. Evaluation of PLA/Silicone Blends Morphology

#### 3.1.1. XRD Results

**Figure 1:**
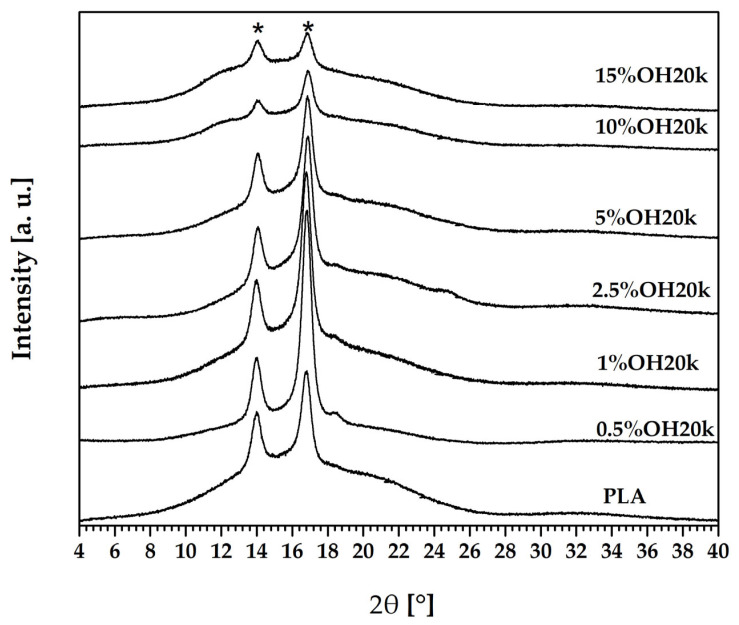
XRD results for the PLA/polysiloxane blends. “*” corresponds to the PLA alpha phase.

To evaluate the crystalline structure of PLA system samples, X-ray diffraction was performed. Diffractograms of the polylactide blends are presented in Figure 1, showing the increasing function of the OH20k content.

The presented polymeric materials exhibit a typical semi-crystalline structure and contain both crystalline regions (shown as visible peaks) and an amorphous “halo” [59,60]. The strongest diffraction peak was observed at 14° and 17° 2θ. They correspond to the (200, 110) reflections of α—PLA [59,61], which is the most stable crystalline structure of PLA [62,63]. The neat PLA pattern shows a slight shift of (200/110) peak positions toward the lower angles. This can be attributed to the low degree of ordering and deformation of the crystal structure [64,65,66]. It can be assumed that neat PLA crystallites adopted the α’ form [59]. The halo derived from the amorphous regions of PLA semicrystalline structure is noticeable for all presented blend compositions (also in neat PLA) and undergoes broadening and flattening due to the function of the sample’s composition. The presence of other crystalline phases is not observed in the XRD patterns, even after the addition of silicone. It is noticed that more significant amounts of OH end-capped silicone in the PLA matrix indicated a reduction in nuclearization effectiveness because of the growing amorphous phase (higher silicone share) in the blend. This can be seen in the decreasing intensity of the reflections as the content of the siloxane additive increases (>10.5%). Similar effects were observed by Yee [67] and Wang [68], as the addition of organic or inorganic silica decreased the degree of crystallinity of the composite. According to Yan [69], the decrease in peak intensity was probably due to the poor crystallization of the macromolecules. Additionally, the small and unstable crystals may have been tightly constrained by the rigid silica network, resulting in a decrease in the crystallinity of the composite. The areas under crystalline and amorphous peaks can be used to calculate the degree of crystallinity [70,71]. PLA and composites with OH20k content were calculated using Formula (2).

Initially, with an increase in silane content, the intensity of the reflections increases (Figure 1) and then begins to decrease. This is reflected in the values of the degree of crystallinity (Table 2). This trend can be observed in the DSC measurements (Table 3). Differences between the obtained results may be due to the different areas of the material being tested. [72]. XRD examines the surface of the sample, while in DSC, the measurement involves the whole volume of sample [73], and the samples from the injection were tested. It should also be noted that the theoretical enthalpy of fusion estimated by Fischer based on Flory’s theory for polymers and copolymers is quite inaccurate when dealing with trans-crystallization in heterogeneous nucleation [57,59].

#### 3.1.2. FTIR Spectra Analysis

**Figure 2:**
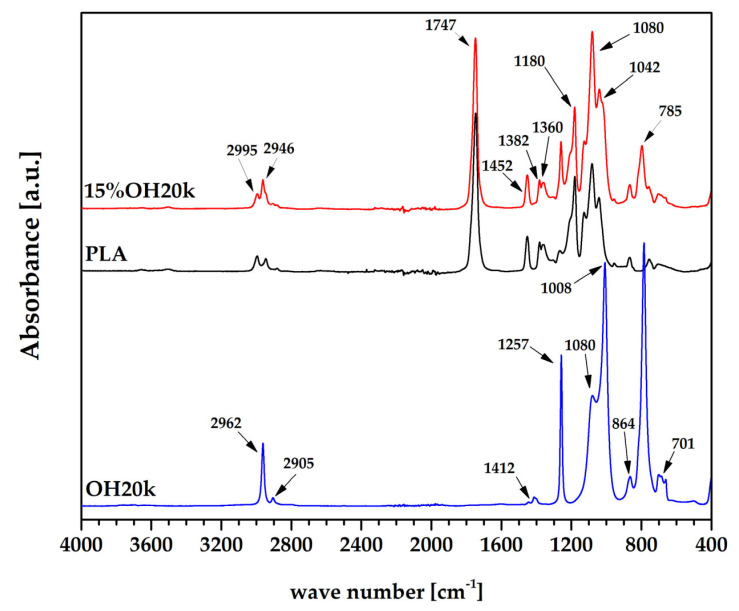
FTIR spectra of PLA, OH20k, and 15% OH20k.

Fourier-transform infrared spectroscopy (FTIR) with ATR analysis was used for structural characterization of the PLA/silicone blend. Figure 2 shows the FTIR spectra of the neat PLA, OH20k, and 15% OH20k samples. Characteristic bands for alkoxypolidimethylsiloxane occur at 701 cm^−1^, 785 cm^−1^ (CH_3_ rocking and Si-C stretching in Si-CH_3_), 864 cm^−1^ (Si-OH stretching), 1080 cm^−1^ (Si-O-Si stretching), 1257 cm^−1^, 1412 cm^−1^ (CH_3_ deformation in Si-CH_3_), 2905 cm^−1^, and 2962 cm^−1^ (CH stretching in CH_3_) [74,75]. The band at 864 cm^−1^ indicates a low content of -OH groups attached to the Si atom capable of condensation, estimated at 600 ppm, according to the manufacturer. Referring to the spectra of neat PLA and its blends, characteristic signals for the PLA structure were noted: 3500 cm^−1^ (-OH stretching), 2995 cm^−1^, 2946 cm^−1^ (-CH stretching), 1747 cm^−1^ (C=O stretching), 1452 cm^−1^, 1382 cm^−1^, 1360 cm^−1^, (CH bending in CH_3_), 1180 cm^−1^ (C-O stretching), 1080 cm^−1^ (C-O-C bending), and 1043 cm^−1^ (C-CH_3_ stretching) [76,77]. The band at the 785 cm^−1^ originating from OH20k Si(CH_3_) appears in 15% OH20k, and is not present in the spectrum of neat PLA. An increase in band intensity at 1080 cm^−1^ due to the overlap of stretching vibrations from Si-O-Si and bending vibrations from C-O-C is also observed.

### 3.2. Composite Structure Evaluation—SEM/MO Observations

#### 3.2.1. Optical Microscopy Observations (MO)

**Figure 3:**
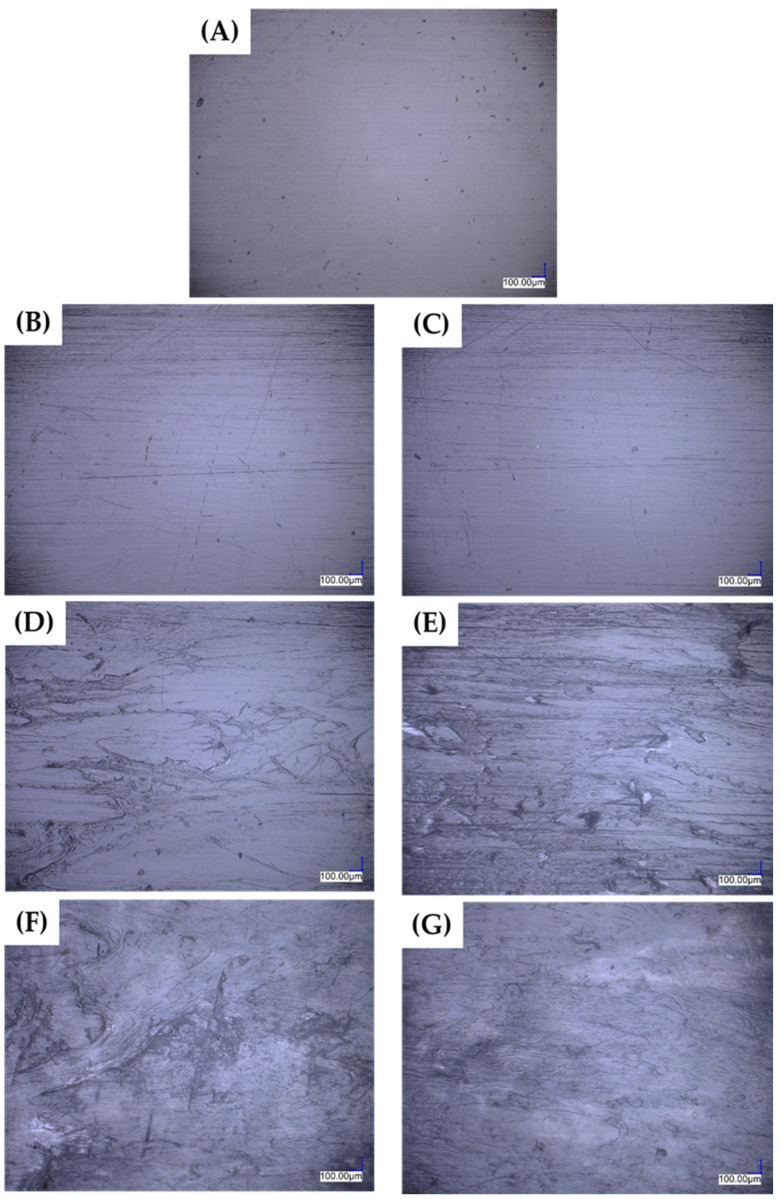
Beam surface topography changes as a function of the OH20k share in the blend: PLA (**A**), 0.5% OH20k (**B**), 1.0% OH20k (**C**), 2.5% OH20k (**D**), 5% OH20k (**E**), 10% OH20k (**F**), 15% OH20k (**G**).

To examine the surface of the specimens and compare the impact of silicone content in PLA blends on their topography and final quality, optical microscopy was performed. Figure 3 shows MO images of PLA and its blends. When it comes to small silicone share (B-C), there is no noticeable contrast in the appearance of the surface shape when compared to PLA (A). Only artifacts in the form of scratches representing the mold surface are visible. The scratches are more pronounced for PLA systems including silicone, which can be explained by an increase in the softness of the material and an increase in the ability to reproduce the shape of the mold cavity, which is linked to changes in the rheological properties of PLA in the presence of silicone (see rheology results 3.4.). An increase in the percentage of silicone in the blend above 2.5% by weight results in a significant deterioration of the surface quality of the produced shapes. On the surface images, we can observe the migration of the diffuse phase to the outer surface of the shape. Silicone migrates to the surface due to the large difference in the viscosity of the blend components, resulting in the formation of crusts and discontinuities, as well as blisters in the surface layers of the test specimens.

#### 3.2.2. Scanning Electron Microscopy (SEM, SEM–EDS)

To evaluate polysiloxane dispersion in the blend and its miscibility with polylactide, SEM images were captured and SEM–EDS analysis were performed. Figure S2 shows the dispersion of phases in the fabricated polymer blends as an increasing function of OH20k concentration in the blends. The surface of PLA is quite smooth, with minimal scratches. However, the addition of the silicone OH20k component influences changes in the surface topology. Polysiloxane is a dispersed phase in the PLA matrix, which is visible in SEM images. Based on microscopic observations, the limited miscibility of polysiloxane and PLA phases can be noticed, especially for higher concentrations, where polysiloxane is enclosed in the PLA structure. SEM–EDS analysis provides information on the distribution of Si on the sample surface, representing the distribution of polysiloxane in the blend structure. It can be concluded that the dispersion for lower polysiloxane weight contents is higher, and the particle distribution is more uniform (Figure S3B,C). The presence of micro-and nano-networks in the PLA/polysiloxane matrices improved the mechanical properties (see Section 3.5). 

Increased impact strength and improved tensile strength were also observed with a low siloxane content. Similar results were achieved by Jeong in his work [78]. The presence of polysiloxanes in the matrix is also responsible for the increase in plasticity, which is caused by the formation of interactions in the PLA matrix, as well as for the increase in flexibility, by slightly reducing the Tg of the polymer [78,79]. With increasing OH20k in the blend, there is a clustering of silicone particles into increasingly larger clusters in the PLA matrix, as we can observe in Figure S3. Meng [80] stated that the higher loading organosilicon molecules have a tendency to form larger aggregates, which have weaker interfacial interactions with the PLA matrix compared to composites with lower modifier content. In addition to the agglomerates of polysiloxane, we can still see a partially well-dispersed phase derived from it. The beginnings of agglomerate formation in the blends (“chicken broth effect”) can already be seen at a 5% OH20k concentration, which is reflected in the lowering of the mechanical properties of the produced blends (see section Mechanical Performance). Micro-sized particles were also observed in the 15% OH20k (Figure S3G). The formation of agglomerates in the blend structure is due to the supersaturation of the system, while the clustering of silicone into spherical dispersion clusters is due to the difference in the hydrophobic–hydrophilic nature of the two blend components. The SEM–EDS analysis confirms previous observations made on SEM and MO images. It can be clearly seen that Si (silicon) is distributed on the PLA surface with a tendency to agglomerate with increasing amounts of polysiloxane. 

### 3.3. Thermal Analysis Results

**Figure 4:**
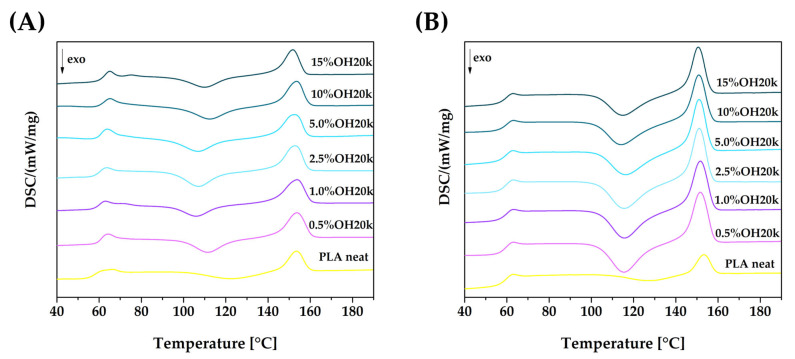
The effects of silicone content on the crystallization and melting behavior of PLA and PLA/silicone blends. DSC curves: first (**A**) and second (**B**) heating cycle.

Based on the DSC analysis of neat PLA and its blends incorporating the polysiloxane (Figure 4), it was observed that the addition of the organosilicon compounds reduces the cold crystallization temperature of the systems (T_cc_ is moved to a lower point position). This is attributed to the nucleation effect under the influence of the additives used, due to an increase in the number of domains favoring the ordering of polylactide chains. Similarly, the melting point of the presented polymer systems also shifts towards lower temperatures.

During the second heating cycle, there is a notable increase in the area under the peak for both T_cc_ and T_m_. The effect of OH20k on PLA crystallization is also discussed in the section on XRD studies (Section 3.1.1). This effect gradually weakens as the silicon content of the blends increases. This can be seen by a decrease in the surface area under the T_cc_ and T_m_ peaks, indicating a reduction in the nucleating nature of the silicone additive for PLA. This is related to the deteriorating miscibility and dispersion of PLA/silicone systems. This is also the effect of an increase in the amorphous phase (silicone-derived) proportion in the PLA/silicone blend. It has been observed that the studied systems show no significant changes in their glass transition or melting temperature as compared to those of the neat polymer (Table 3).

Thermograms of the modified PLA compositions are shown in Figure S4, and thermogravimetric analysis data are summarized in Table 4. With a 5% weight loss, a slight shift towards higher temperatures was observed, with a maximum of 8.2 for the 5% polysiloxane content. Neither the temperature at the beginning of thermal decomposition nor the temperature at the maximum mass loss rate show any significant deviations from the reference sample. For systems containing 10% and 15% polysiloxane, the occurrence of additional T_onset_ and T_max_ peaks was reported, which result from silicone and are related to the decomposition of its main Si-O-Si chain, which decomposes at higher temperatures than does the carbon polymer chain [47]. The presence of additional temperatures T_onset_ and T_max_ confirms the decrease in the mutual compatibility of both polymers. The lack of significant changes in the values of these temperatures indicates the lack of influence of polysiloxane on the mechanisms of the thermal decomposition of polylactide.

The HDT measurement was used to measure the ability of PLA and its blends to withstand force (during three-point bending) on a material sample exposed to elevated temperatures. This is an easily measured thermal parameter important for designers during the modeling of mechanical parts in order to determine short-term heat resistance under force. Analyzing the bending behavior, it was observed that incorporating a high molecular weight organosilicon polymer in the polymer blend system led to a stiffness increase of up to 5% OH. It is worth noting that this phenomenon persisted, even at high temperatures, resulting in a minor increase in the HDT temperature of PLA/silicone compositions (Table 5). However, differences between compositions were almost imperceptible until the OH20k content increases to 10%. The improvement in HDT values is correlated with the crystallinity level of the material’s compositions.

The crystallinity level was calculated based on the DSC data obtained from the first heating cycle. Based on *X_c_* results, the nucleating character changes in the silicone used in the blend are shown. A small area showing PLA’s cold crystallization peak on the DSC thermograms indicates PLA’s low capacity to crystallize under the cooling conditions applied during the injection molding process, which is typical of many species of PLA. The incorporation of even small quantities of OH20k enhances the rate of crystal structure formation in the blended material; thus, it may be concluded that it is a highly effective nucleating agent (100% *X_c_* increase related to neat PLA). It has also been noticed that crystallinity levels do not change significantly between characterized systems.

### 3.4. Rheology

**Figure 5:**
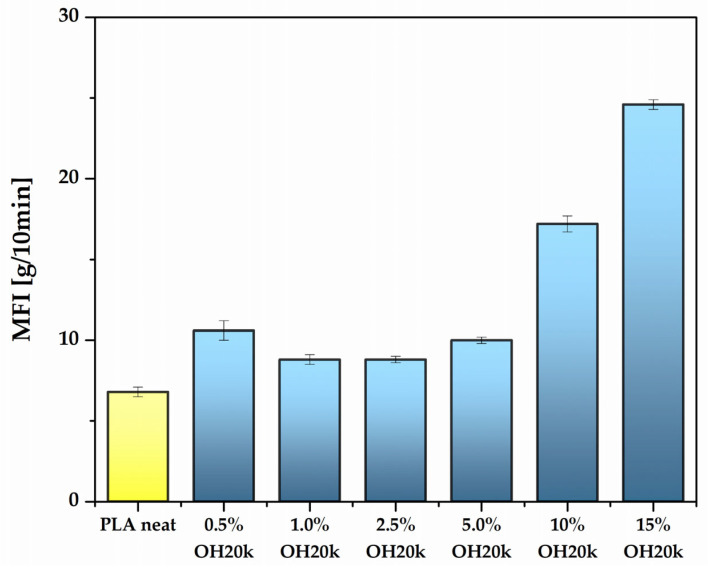
Effect of silicone content on the technological properties of PLA.

The results of the melt flow rate (MFI) analysis of the obtained mixture samples are shown in Figure 5. The MFI of pure PLA is approximately 7.0 g/10 min. It was observed that the addition of polysiloxane to the PLA matrix causes a significant increase in the flowability of the composite. For 15% OH20k, the increase in MFI compared to that of pure PLA is almost 275%. This phenomenon is attributed to the plasticizing effect of the additive in use. As per the lubricity theory, the plasticizer serves to lubricate the PLA chains, thereby improving the processability of the PLA/silicone blends by minimizing friction between the chains and facilitating easier sliding between them [81]. Polysicoxane contains hydroxyl groups in its structure, capable of forming weak chemical interactions with PLA and acting as a self-lubricant, results which correspond to those from our previous observations [11]. Improvements in mixture processing were already observed during the sample preparation process using injection molding.

### 3.5. Mechanical Performance

#### 3.5.1. Flexural Behavior Analysis

**Figure 6:**
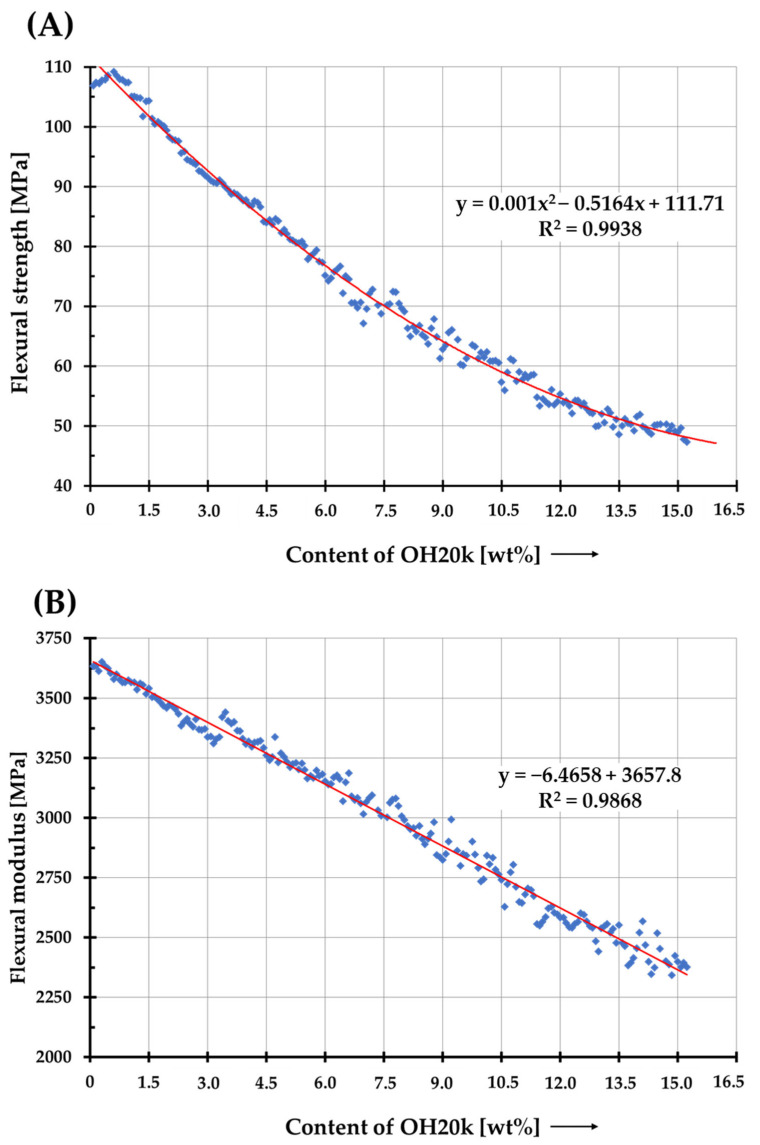
Flexural characteristics of PLA and its silicone blends as a function of silicone content (0–15%), obtained by the robot. Flexural strength (**A**); flexural stiffness (**B**).

In applications where materials or parts are subjected to high pressure or stress, possessing high flexural strength is crucial for effective stress transfer. Furthermore, the flexural strength of a material determines its usability in certain situations. Measuring flexural modulus is an essential aspect of engineering, as it enables the gauging of a material’s ability to resist bending. The bending properties of the developed mixtures were comprehensively characterized by measuring the elastic modulus and bending strength while continuously changing the concentration of polysiloxane in the PLA matrix. After collecting data from multiple measurement points, the established trend curve can be used as a calibration or selection curve. Stress–deflection curves for all samples show changes in the bending behavior of PLA/silicone blends, depending on the weight proportions of PLA and silicone (Figure S5). As the weight content of polysiloxane increases, the modulus of stiffness of the blend decreases, which is related to an increase in the elasticity of rigid PLA in the presence of the additive. Polysiloxanes, due to their chemical structure, exhibit higher molecular mobility than PLA and act as plasticizers. By infiltrating the molecular chains of PLA, polysiloxane increases the free spaces in the polymer matrix, reducing intramolecular friction between its macromolecules and allowing them to move freely past each other. These results are in agreement with the rheological analysis and microscopic observations. Based on the results (Figure 6), it can be concluded that the bending stiffness, like the bending strength, follows a linear trend curve as a function of the OH20k concentration. At low silicone concentrations in PLA systems, an improvement in bending strength is observed (16% increase in the case of pure PLA).

Common practice in science involves presenting the results based on a narrowly defined group of concentrations of modifiers, fillers, additives, etc. used for plastics. However, the selection of these components is often unclear and lacks transparency. Through analysis of various individual measurement points, such as the concentration of the additive used, the way in which the material will behave during the analysis is predicted. The results are presented in the context of specific ranges. Based on three-point bending tests performed in manual mode (Table 6), it can be concluded that with a small number of measurement points, it is difficult to properly adjust the trend curve to the obtained results and conclude from them how the material will behave in a certain range of values. The differences are particularly noticeable when it comes to the low concentrations of OH20k silicone found in PLA systems.

#### 3.5.2. Static Tensile Behavior and Impact Resistance Results

For comparison with the three-point bending tests, the concentration of OH20k silicone in the PLA blend was selected for further analysis of its mechanical properties. Additional important mechanical properties are presented in Table 6. As was noted previously, the addition of OH20k silicone significantly changed the mechanical properties of the PLA, and the impact behavior of the presented samples is related to their material structure and the proportions of the components in the blend.

The nucleating effect caused by silicone increases the degree of crystallinity and stiffness moduli due to a higher nucleation density and an increase in order in the PLA structure [82]. The effect of nucleation is superimposed with additional effects resulting from the plasticizing properties of the silicone forming the blend. Tensile strength is decreased due to higher contents of OH20k silicone, which is linked to the softening of PLA [83]. Supersaturation of the PLA/silicone system and phase separation are common occurrences when there is a significant difference in surface properties between polymer blend phases [84]. In addition, SEM observations have revealed that excessive silicone leads to the formation of a separate phase, resulting in a lack of homogeneity in the composition and lower tensile strength values.

The elongation at break of neat polylactide is determined as nearly 6%, which is consistent with the literature data. The 2.5% OH20k sample reveals the ε value of 19%, which is 225% more than that of neat PLA. The high values in regards to the samples breaking strain under the influence of the axially applied static tensile force are clearly due to the strong plasticizing effect of OH20k [85]. The addition of up to 5% OH20k resulted in the reduction of ε values. An amount of 5% by weight of silicone in PLA can be considered as the point above which the miscibility of the phases in the blend drops drastically. The elongation at break noticeably increases past this point.

PLA is known as a brittle material with low impact resistance. The Charpy impact strength of neat PLA is around 17.5 kJ/m^2^ for the Ingeo 2003D type. As noted in the previously presented microscopic analysis, silicone is a dispersed phase in the blend (particle-like filler). This behavior is caused by the arrangement of PLA macromolecules on small silicon particles (nucleation), as well as by the plasticization of the polymer matrix. The presence of small particles of silicone, which have a much lower viscosity than PLA, can improve the mobility of the PLA macromolecule chains. Thus, more energy is consumed during the impact. Moreover, the blend samples are characterized by higher degree of crystallinity than neat PLA, which can also influence their impact resistance [86]. It should also be noticed that in the case of impact strength, the trend in the influence of OH-silicone is similar to that of the elongation properties. The decrease in impact strength below the PLA reference value (5% OH20k) can be attributed to the non-homogeneous structure of PLA/silicone blend systems. Any discontinuities in the structure can cause cracks to form.

## 4. Conclusions

The work presents an innovative approach to measuring the mechanical properties of materials using an automated and robotic measurement station, which allowed for the precise development of a high-performance auxiliary tool. A thorough investigation was conducted regarding how polydimethylsiloxane terminated with OH groups affects the properties of polylactide. The study confirmed that silicone is an effective nucleating additive to polylactide, as shown by the increase in the degree of crystallinity, based on DSC measurements and XRD diffractograms. The addition of polysiloxane resulted in the improved mechanical properties of PLA blends. This led to an increase in impact strength, stiffness, and deformation at break. These results were due to both its plasticizing properties and its ability to increase the degree of crystallinity of the material. However, the study also found that the miscibility of silicone with PLA significantly decreased beyond certain limits, resulting in a deterioration of the mechanical properties of the blends. Further research is needed to improve the miscibility of the systems. No significant influence of silicone on the thermal properties of PLA has been proven.

## Figures and Tables

**Table 1:** Sample designation and material formulations for the prepared polymer blends.

Sample Abbreviation	PLA	Silicone
PLA neat	100	-
0.5% OH20k	99.5	0.5
1.0% OH20k	99	1.0
2.5% OH20k	97.5	2.5
5.0% OH20k	95	5.0
10% OH20k	90	10
15% OH20k	75	15

**Table 2:** Degree of crystallinity PLA and its composites with OH20k, based on XRD.

Sample	*X_c_*, %
PLA neat	30, 49
0.5% OH20k	31, 23
1.0% OH20k	32, 37
2.5% OH20k	31, 68
5.0% OH20k	32, 15
10% OH20k	30, 58
15% OH20k	27, 88

**Table 3:** Results of differential scanning calorimetry analysis (N2) from the second heating cycle.

	Glass Transition Temperature, T_g_ [°C]	Cold Crystallization Temperature, T_cc_ [°C]	Melting Temperature, T_m_ [°C]
PLA neat	63.2	127.2	155.4
0.5% OH20k	62.7	115.4	151.7
1.0% OH20k	62.9	115.9	151.7
2.5% OH20k	62.7	115.6	151.2
5.0% OH20k	62.6	115.7	151.1
10% OH20k	62.8	114.0	151.8
15% OH20k	62.2	114.7	150.8

**Table 4:** Results of thermogravimetric analysis (N2).

	5% Mass Loss Temperature, T_5%_ [°C]	Onset Temperature, T_onset_ [°C]	Temperature of Maximum Mass Loss Rate, T_max_ [°C]
PLA neat	324.2	342.2	362.1
0.5% OH20k	328.6	343.8	362.7
1.0% OH20k	329.2	344.2	363.4
2.5% OH20k	330.5	347.4	364.6
5.0% OH20k	332.0	349.4	361.9
10% OH20k	332.4	345.3, 520.6	364.7, 550.4
15% OH20k	327.9	343.8, 478.0	361.2, 507.40

**Table 5:** Heat distortion temperature (HDT) and crystallinity level of PLA and its blends.

Sample Abbreviation	HDT [°C]	*X_c_* [%]
PLA neat	55.80 ± 0.10	16.44
0.5% OH20k	57.16 ± 0.06	34.24
1.0% OH20k	57.23 ± 0.06	34.04
2.5% OH20k	57.17 ± 0.06	34.62
5.0% OH20k	57.10 ± 0.00	32.27
10% OH20k	52.73 ± 0.06	36.51
15% OH20k	52.60 ± 0.10	32.48

**Table 6:** Mechanical properties (flexural, tensile, impact) of PLA-based test specimens.

Sample Abbreviation	Flexural Strength [MPa]	Flexural Modulus [GPa]	Tensile Strength [MPa]	Young’s Modulus [GPa]	Elongation at Break [%]	Impact Resistance [kJ/m^2^]
PLA neat	95.60 ± 0.94	3.52 ± 0.03	60.61 ± 0.42	2.69 ± 0.22	6.25 ± 0.54	17.36 ± 0.47
0.5% OH20k	95.87 ± 0.85	3.75 ± 0.05	55.29 ± 0.41	3.36 ± 0.03	10.09 ± 1.69	17.81 ± 1.20
1.0% OH20k	92.33 ± 1.23	3.62 ± 0.09	53.03 ± 3.62	3.28 ± 0.04	13.07 ± 3.19	20.36 ± 2.81
2.5% OH20k	85.85 ± 2.07	3.75 ± 0.06	52.43 ± 0.80	3.33 ± 0.03	19.40 ± 3.60	27.97 ± 3.52
5.0% OH20k	74.40 ± 0.33	3.58 ± 0.03	45.93 ± 0.72	3.05 ± 0.08	10.29 ± 2.69	24.23 ± 2.88
10% OH20k	54.19 ± 3.81	3.27 ± 0.05	41.24 ± 0.22	2.79 ± 0.02	2.57 ± 0.22	12.46 ± 1.66
15% OH20k	40.59 ± 2.85	2.78 ± 0.04	33.91 ± 0.37	2.42 ± 0.02	2.18 ± 0.20	10.87 ± 2.58

## Data Availability

Data are contained within the article and supplementary materials.

## Supplementary Materials

The following supporting information can be downloaded at: https://www.mdpi.com/article/10.3390/polym16081142/s1, Figure S1: High-throughput experimentation station: Dobot Magician manipulator (1), linear slide (2), sample magazine (3), and MTS Insight testing machine (4). Figure S2: SEM images PLA (A), 0.5%OH20k (B), 1.0%OH20k (C), 2.5%OH20k (D), 5%OH20k (E), 10%OH20k (F), 15%OH20k (G). Figure S3: Si (silicon) distribution on the surface of PLA/OH20k blends breakthroughs made in liquid nitrogen. 0.5%OH20k (A), 1.0%OH20k (B), 2.5%OH20k (C), 5%OH20k (D), 10%OH20k (E), 15%OH20k (F). Figure S4: The effects of silicone content on the thermal decomposition of PLA and PLA/silicone blends: DTG (A), TGA (B)—N2 atmosphere. Figure S5: Stress-deflection behavior of PLA and its blends as a function of silicone content (0-15%) derived from automatic test station. Table S1: Injection molding parameters.
