# Supplementary material for: OH End-Capped Silicone as an Effective Nucleating Agent for Polylactide—A Robotizing Method for Evaluating the Mechanical Characteristics of PLA/Silicone Blends
Source: Polymers (Basel). 2024 Apr 18;16(8):1142. doi: 10.3390/polym16081142 (PMC11053881; doi:10.3390/polym16081142)
Supplement: Supplementary file 1 [file polymers-16-01142-s001.zip › polymers-2920540-supplementary.pdf]

Supplementary data for Polymers

OH End-Capped Silicone as an Effective Nucleating Agent for Polylactide—A Robotizing Method for Evaluating the Mechanical Characteristics of PLA/Silicone Blends

Robert E. Przekop [1], Bogna Sztorch [1,*], Julia Głowacka [1,2], Agnieszka Martyła [1], Eliza Romańczuk-Ruszuk [3], Marek Jałbrzykowski [4] and Łukasz Derpeński [4,*]

[1] Centre for Advanced Technologies, Adam Mickiewicz University in Poznań, 10 Uniwersytetu Poznańskiego, 61-614 Poznań, Poland; rprzekop@amu.edu.pl (R.E.P.); julia.glowacka@amu.edu.pl (J.G.); agnieszka.martyla@amu.edu.pl (A.M.)
[2] Faculty of Chemistry, Adam Mickiewicz University in Poznań, 8 Uniwersytetu Poznańskiego, 61-614 Poznań, Poland
[3] Institute of Biomedical Engineering, Faculty of Mechanical Engineering, Bialystok University of Technology, Wiejska 45C Street, 15-351 Bialystok, Poland; e.romanczuk@pb.edu.pl
[4] Institute of Mechanical Engineering, Faculty of Mechanical Engineering, Bialystok University of Technology, Wiejska 45C Street, 15-351 Bialystok, Poland; m.jalbrzykowski@pb.edu.pl
* Correspondence: bogna.sztorch@amu.edu.pl (B.S.); l.derpenski@pb.edu.pl (Ł.D.)

1. Figures
1.1. Figure S1…………………………………………………………………………………… 2
1.2. Figure S2………………………………………………………………………………….... 3
1.3. Figure S3…………………………………………………………………………………… 7
1.4. Figure S4…………………………………………………………………………………… 8
1.5. Figure S5…………………………………………………………………………………… 9
2. Tables
2.1. Table S1…………………………………………………………………………………….. 9

1. Figures

1.1. Figure S1

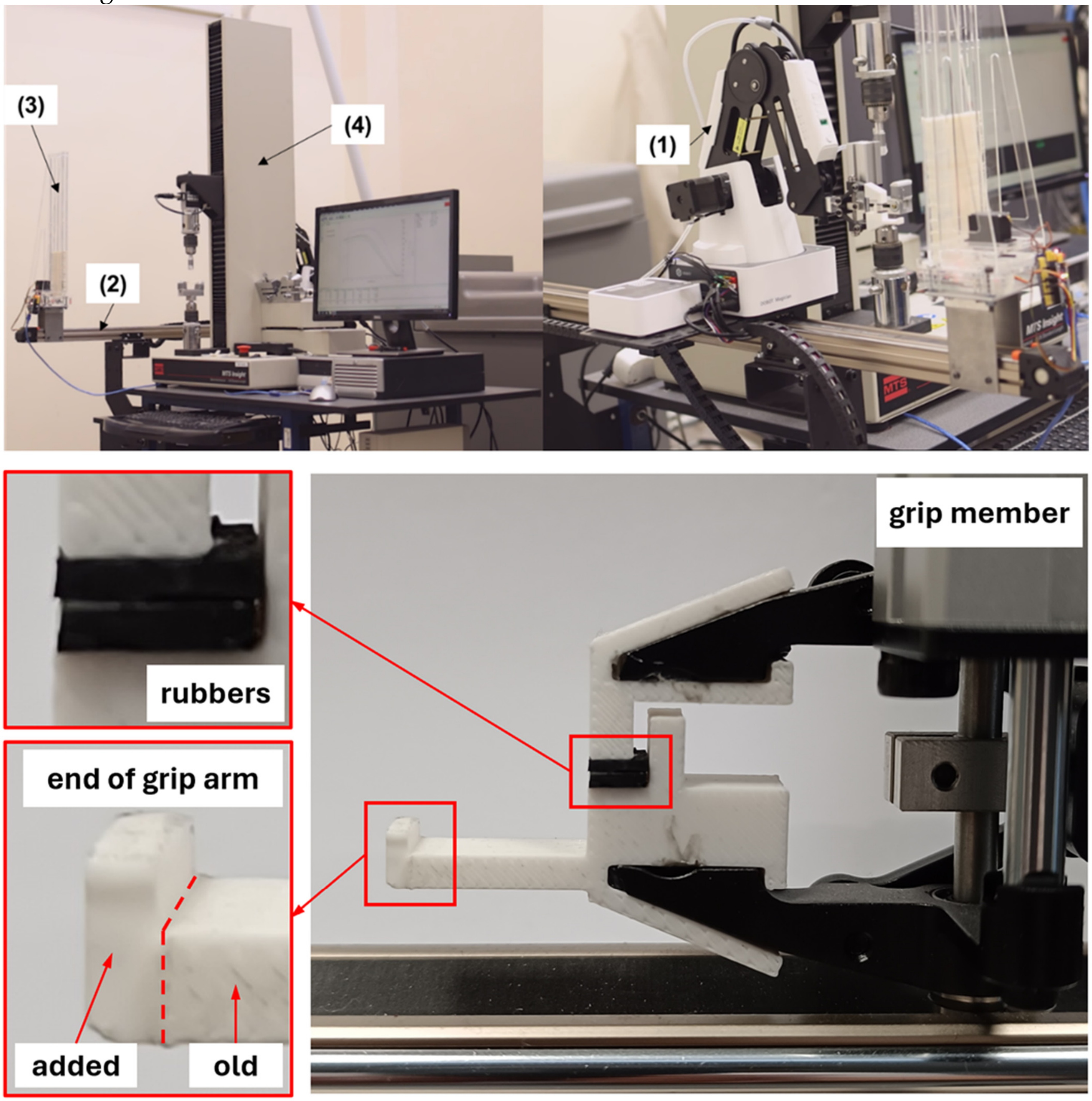

Figure S1. High-throughput experimentation station: Dobot Magician manipulator (1), linear slide (2), sample magazine (3), and MTS Insight testing machine (4).

1.2. Figure S2

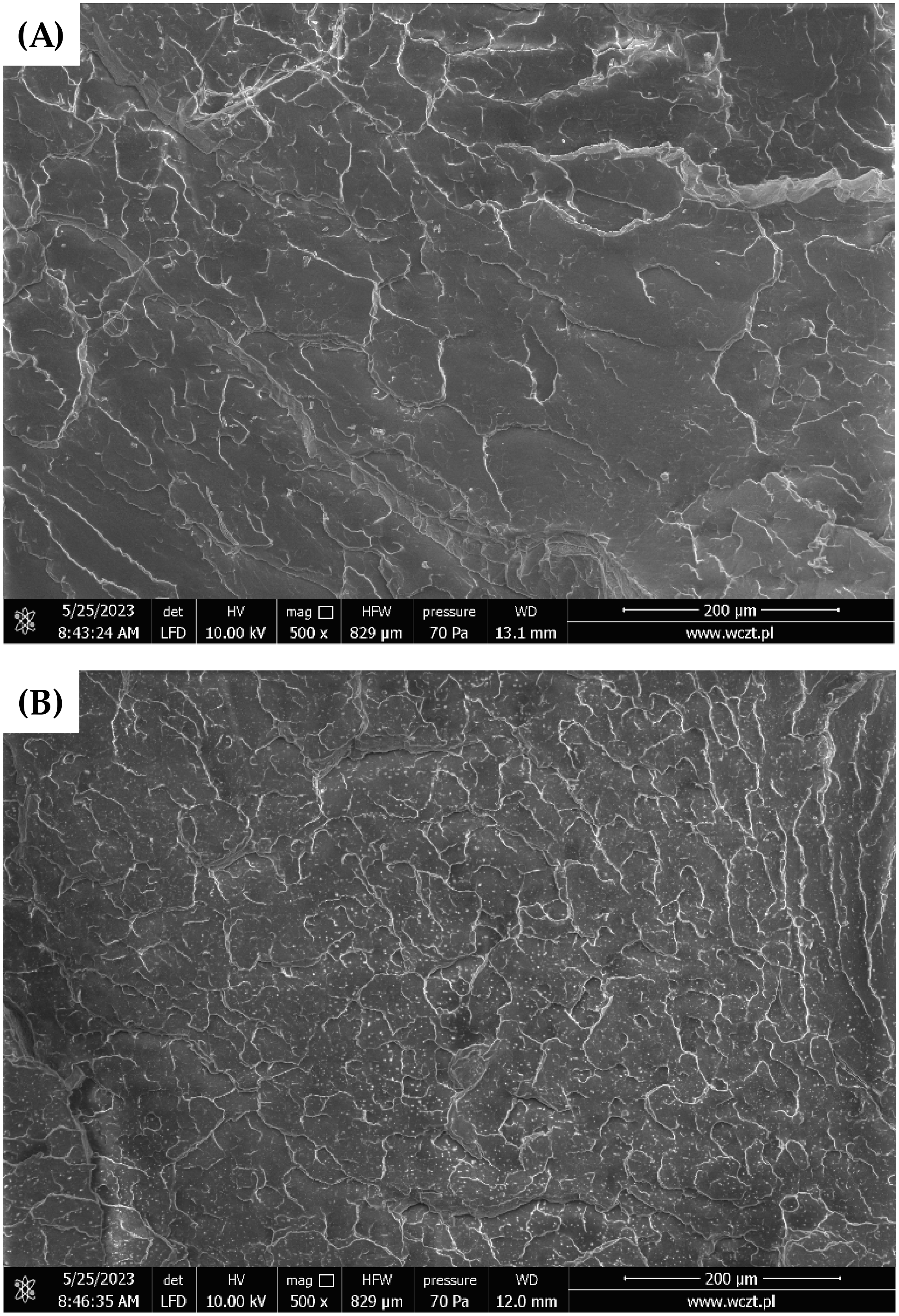
(A)
5/25/2023
8:43:24 AM
det
LFD
HV
10.00 kV
mag
500 x
HFW
829 μm
pressure
70 Pa
WD
13.1 mm
200 μm
www.wczt.pl
(B)
5/25/2023
8:46:35 AM
det
LFD
HV
10.00 kV
mag
500 x
HFW
829 μm
pressure
70 Pa
WD
12.0 mm
200 μm
www.wczt.pl

(C)

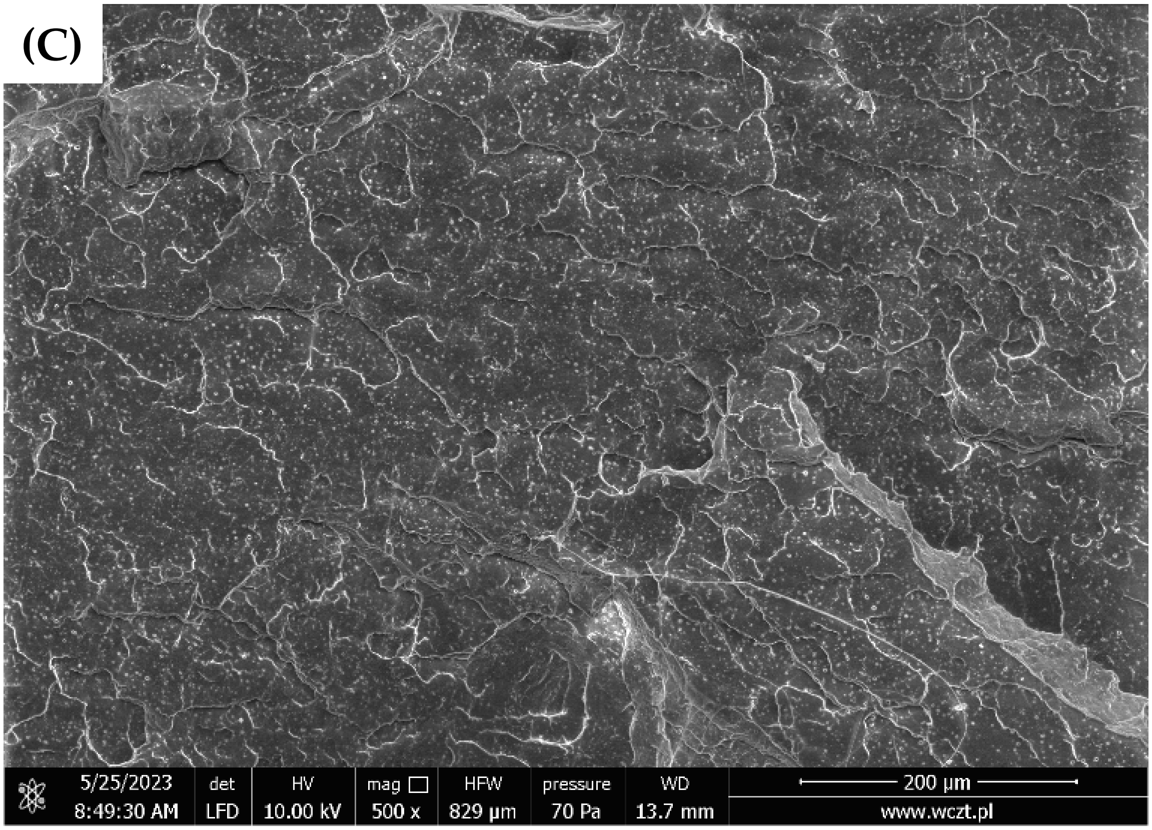
5/25/2023
8:49:30 AM
det
LFD
HV
10.00 kV
mag
500 x
HFW
829 µm
pressure
70 Pa
WD
13.7 mm
200 µm
www.wczt.pl

(D)

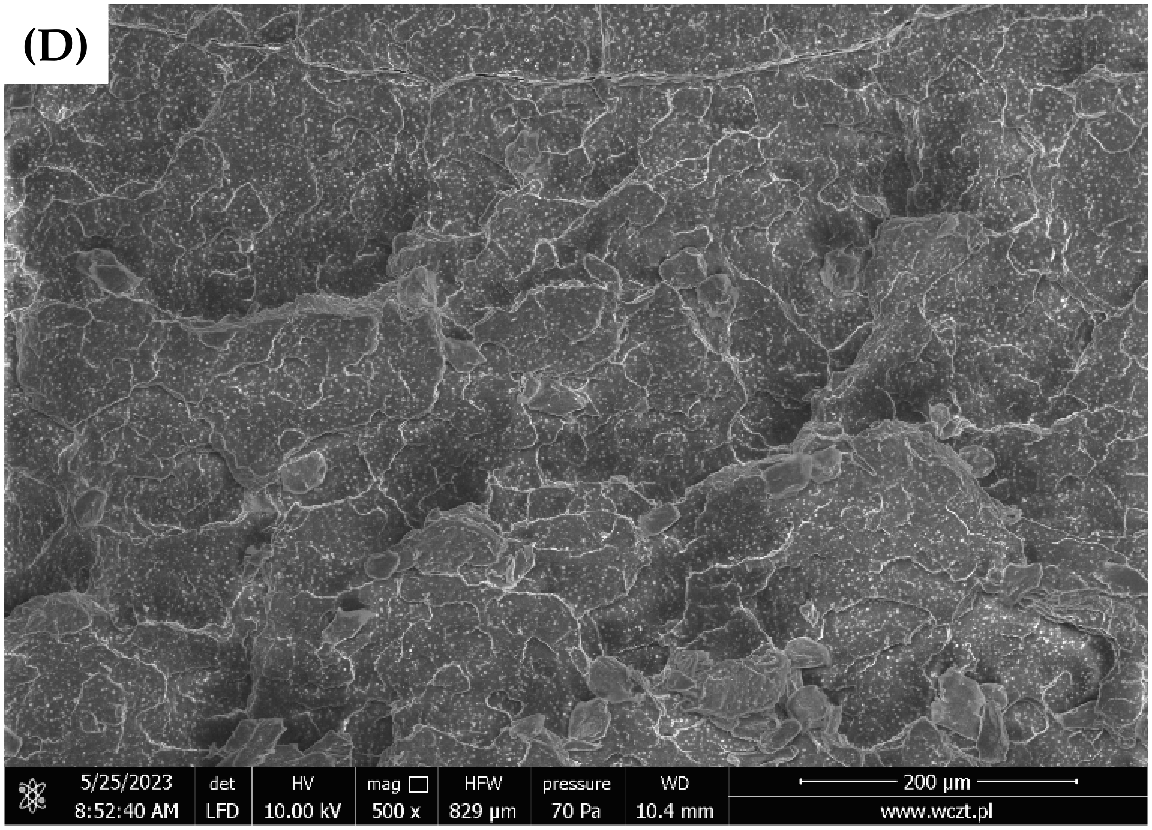
5/25/2023
8:52:40 AM
det
LFD
HV
10.00 kV
mag
500 x
HFW
829 µm
pressure
70 Pa
WD
10.4 mm
200 µm
www.wczt.pl

(E)

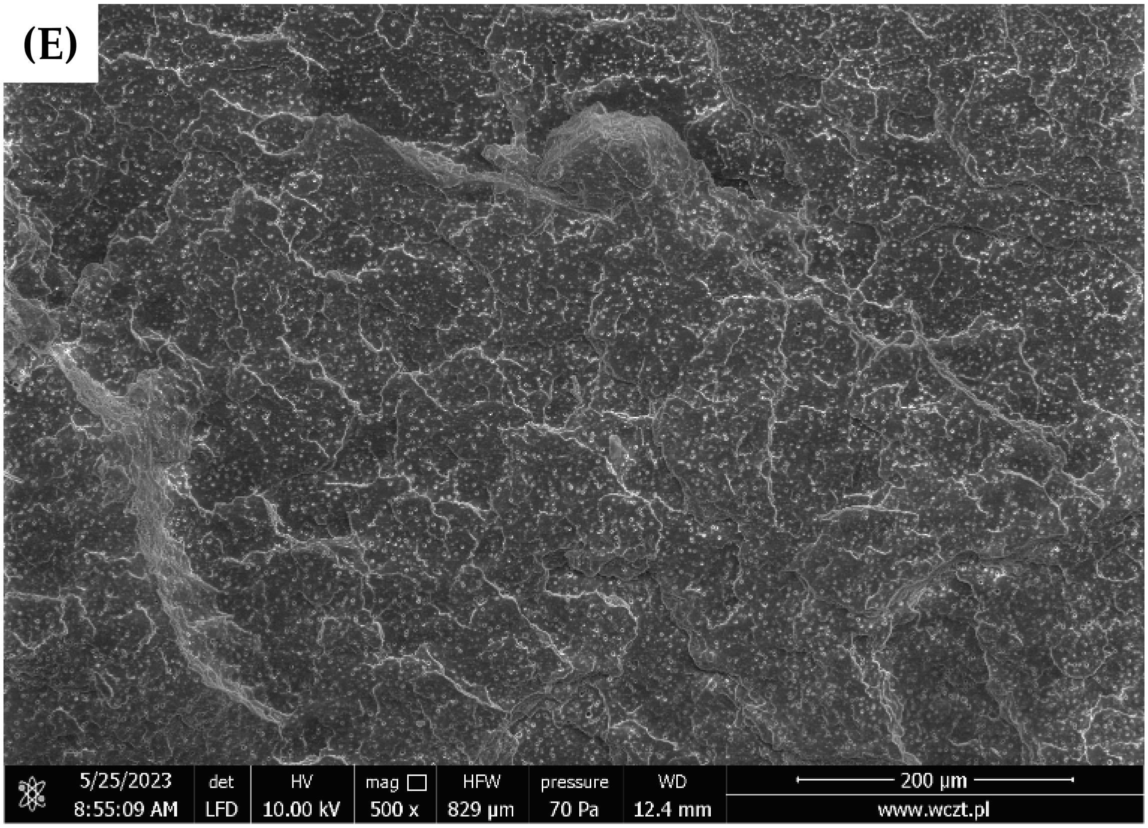
5/25/2023
8:55:09 AM
det
LFD
HV
10.00 kV
mag
500 x
HFW
829 µm
pressure
70 Pa
WD
12.4 mm
200 µm
www.wczt.pl

(F)

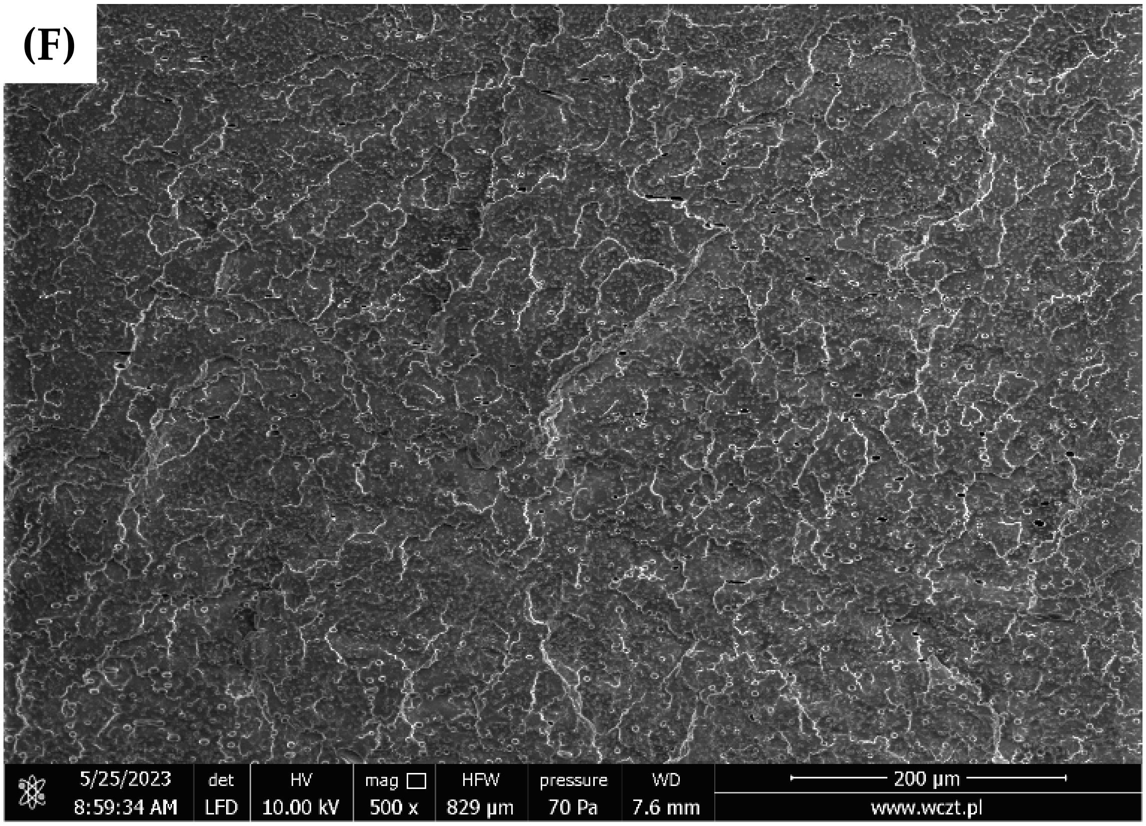
5/25/2023
8:59:34 AM
det
LFD
HV
10.00 kV
mag
500 x
HFW
829 µm
pressure
70 Pa
WD
7.6 mm
200 µm
www.wczt.pl

(G)

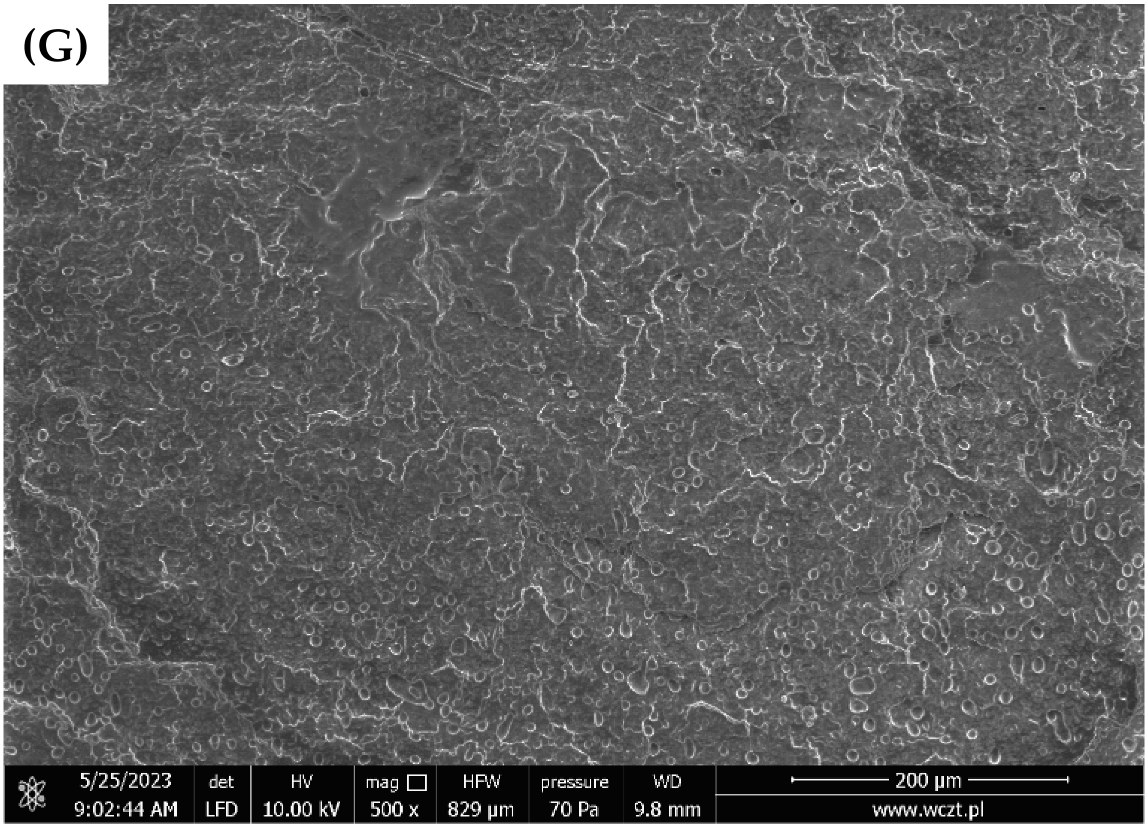

Figure S2. SEM images PLA (A), 0.5%OH20k (B), 1.0%OH20k (C), 2.5%OH20k (D), 5%OH20k (E), 10%OH20k (F), 15%OH20k (G).

1.3. Figure S3

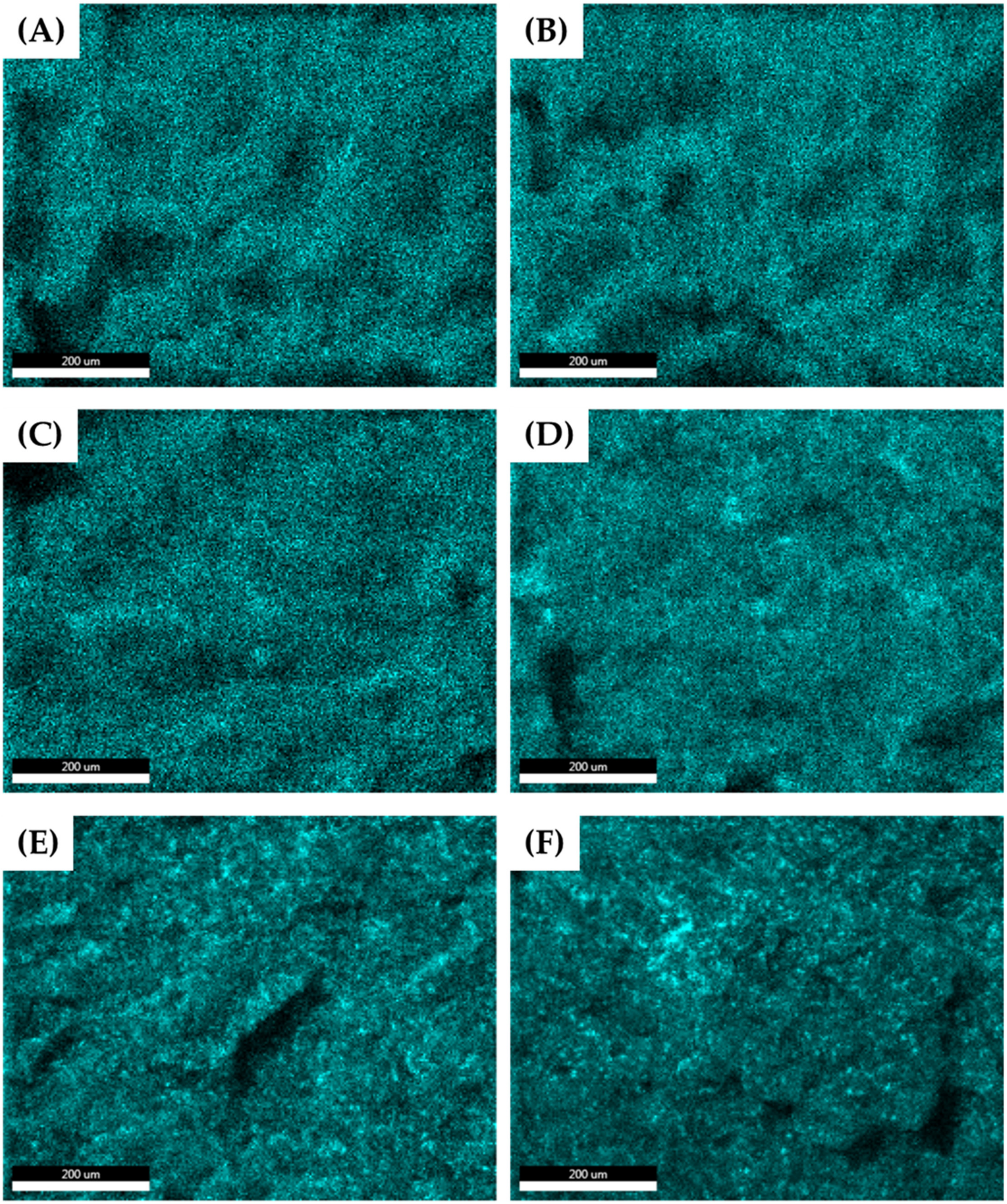

Figure S3. Si (silicon) distribution on the surface of PLA/OH20k blends breakthroughs made in liquid nitrogen. 0.5%OH20k (A), 1.0%OH20k (B), 2.5%OH20k (C), 5%OH20k (D), 10%OH20k (E), 15%OH20k (F).

1.4. Figure S4

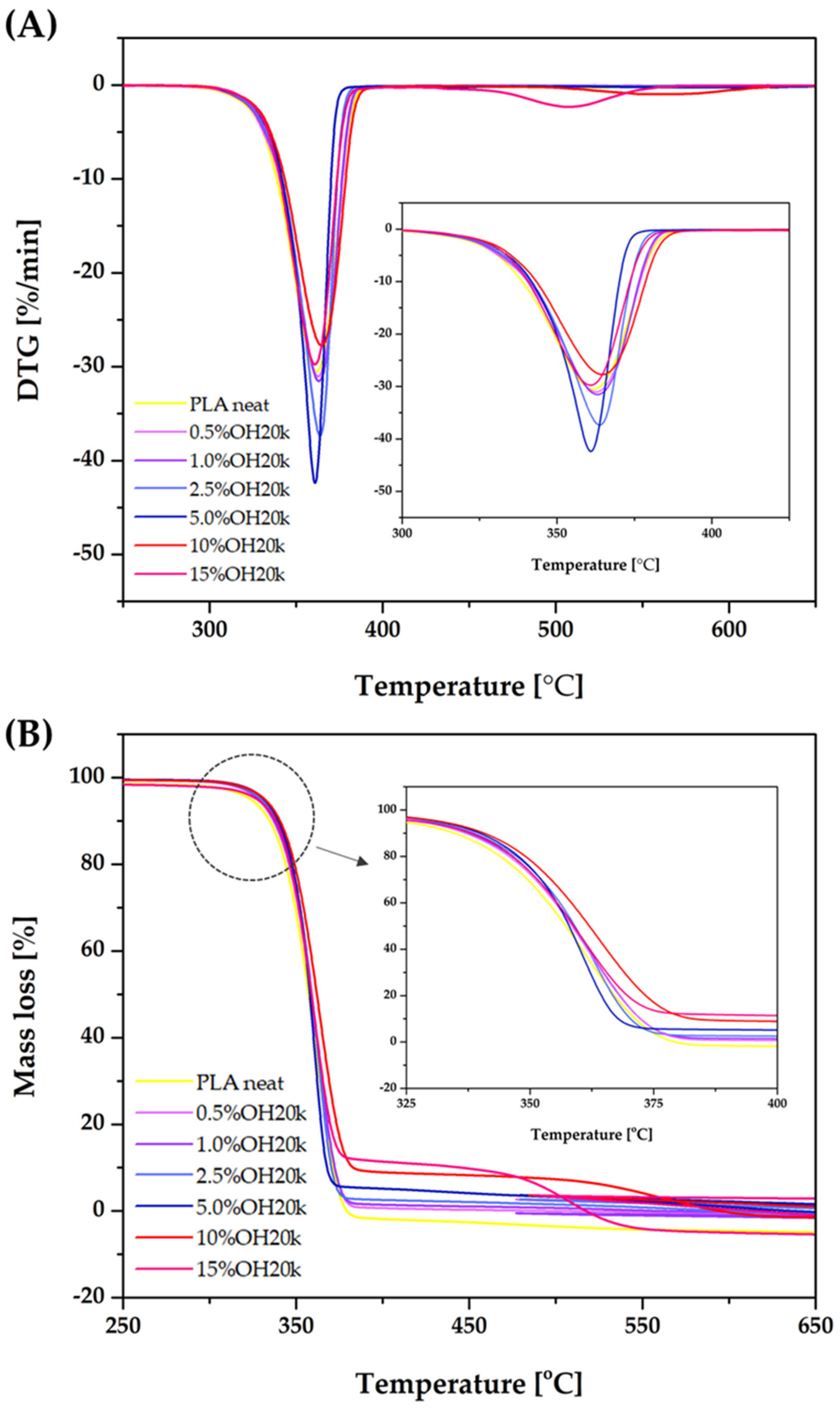

Figure S4. The effects of silicone content on the thermal decomposition of PLA and PLA/silicone blends: DTG (A), TGA (B) – N2 atmosphere.

1.5. Figure S5

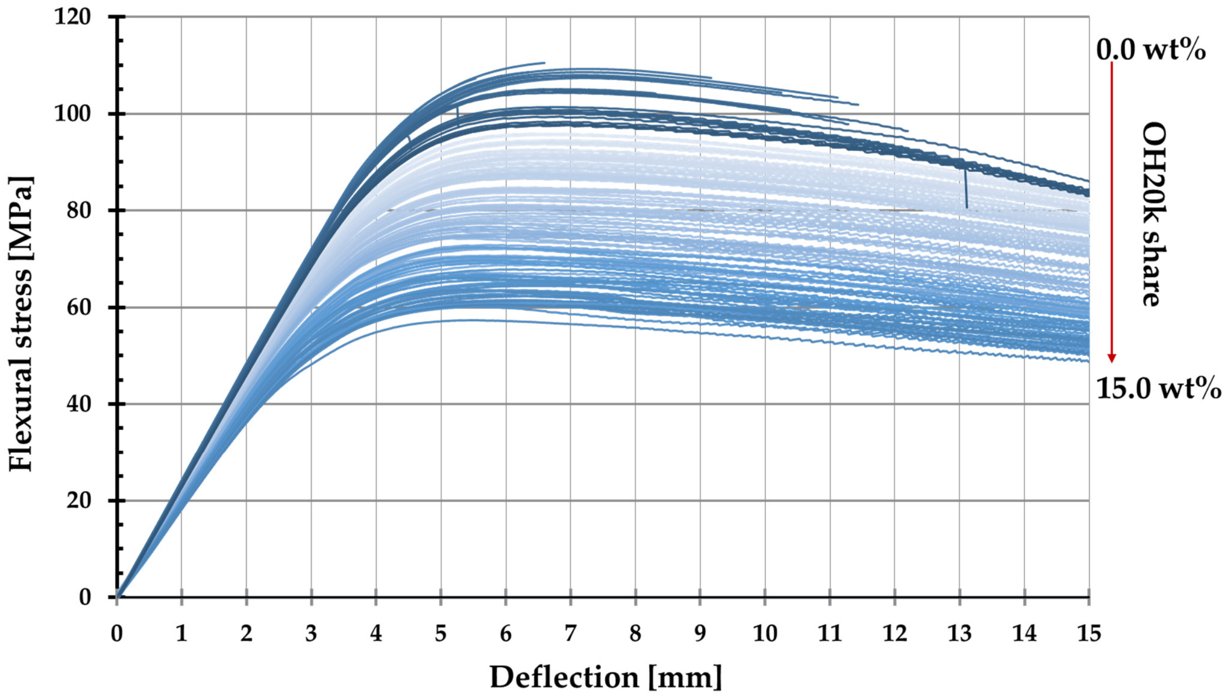

Figure S5. Stress-deflection behavior of PLA and its blends as a function of silicone content (0-15%) derived from automatic test station.

2. Tables

2.1. Table S1

Table S1. Injection molding parameters.

**Temperature (◦C)**	**Nozzle**	**Zone 3**	**Zone 2**	**Zone 1**	**Feed**
	200	195	195	190	40
Mold temperature (◦C)	25				
Holding pressure	t (s)	0	11		
	p (bar)	700	1100		
Clamping force (kN)	Holding pressure time (s)	Cooling time (s)	Screw diameter (mm)		
800	11	50	25		
